# Gender equity and COVID-19 vaccine policies for pregnant people: a global analysis

**DOI:** 10.1186/s12939-025-02497-0

**Published:** 2025-05-07

**Authors:** Eleonor Zavala, Elizabeth Doggett, Andrew Nicklin, Ruth A. Karron, Ruth R. Faden

**Affiliations:** 1 Department of International Health, Bloomberg School of Public Health, Johns Hopkins University, 615 N. Wolfe St, Baltimore, MD 21205 USA; 2 Jhpiego, 1615 Thames Street, Baltimore, MD 21231 USA; 3 Bloomberg Center for Government Excellence, Johns Hopkins University, 711 W 40th St, Baltimore, MD 21211 USA; 4 Berman Institute of Bioethics, Johns Hopkins University, 1809 Ashland Avenue, Baltimore, MD 21205 USA

**Keywords:** Gender equity, COVID-19 vaccine, Pregnancy, Bioethics, Health policy

## Abstract

**Background:**

Despite increasing vaccine availability and evidence and expert recommendations to support administration, some countries maintained restrictive policies regarding COVID-19 vaccination in pregnancy throughout the pandemic. This global analysis explores the role of gender equity, country income level, and vaccine availability in predicting national policies on COVID-19 vaccine administration in pregnancy.

**Methods:**

Policies were collected from May 2021 to January 2023 from 224 countries/territories using publicly available information posted on national public health authority web pages. Policies were categorized into 6 types, representing different levels of permissiveness, from recommended for some or all to not recommended, and changes in national policies were captured over time. Outcomes were defined as: 1) prevalence of restrictive policies at a specific time point; 2) country-level change from restrictive policy/no position at an earlier time point to a permissive policy at a later timepoint. Simple and multivariable logistic regressions were performed to explore the association between the outcomes and potential policy predictors, including income level, mRNA vaccine availability, and the Global Gender Gap Index (GGGI).

**Results:**

Complete cross-sectional data were available for 114 countries as of June 2021, 137 countries as of October 2021, and 142 countries as of March 2022. The number of maternal immunization policies increased and became steadily more permissive between 2021 and 2022. Availability of mRNA vaccines and higher income level were associated with reduced odds of a restrictive policy at the 2021 timepoints, and higher GGGI scores were associated with reduced odds of restrictive policies at all timepoints. After adjusting for income level and mRNA vaccine availability, higher GGGI scores reduced the relative odds of a restrictive COVID-19 vaccine policy by 10% (aOR: 0.90, 95CI: 0.81, 0.99) in October 2021 and 14% (aOR: 0.86, 95%CI: 0.76, 0.97) in March 2021. Higher GGGI scores were also associated with increased odds of a policy switch from restrictive/no position in June 2021 to permissive in October 2021 (aOR: 1.12, 95%CI: 1.00, 1.24).

**Conclusions:**

Gender inequity was associated with greater odds of a restrictive policy for use of COVID-19 vaccines in pregnancy, suggesting that gender biases may influence fair policymaking for pregnant people in pandemic preparedness and response.

**Supplementary Information:**

The online version contains supplementary material available at 10.1186/s12939-025-02497-0.

## Background

As COVID-19 vaccines were licensed and authorized in late 2020 and early 2021, policymakers developing recommendations for use of COVID-19 vaccines were challenged by the absence of efficacy and safety data in pregnancy. In response, countries took a variety of positions on COVID-19 vaccine administration in pregnant persons, from broadly restrictive to largely permissive. To inform how policies were shaping access to vaccines in pregnancy, and the influences affecting policy, our group developed the COVID-19 Maternal Immunization Tracker (COMIT). COMIT captured and recorded the evolution of national policies on the use of COVID-19 vaccines in pregnant and lactating people between May 2021 and January 2023 (www.comitglobal.org).

Findings from this initiative demonstrated how shifts toward more permissive policies occurred as data emerged about the substantial risks of COVID-19 during pregnancy, including increased maternal mortality and adverse birth outcomes [1–3], and as evidence mounted on the safety and benefits of vaccination during pregnancy, providing protection against severe illness for mother and newborn [4–6]. We noted that global shifts in policy aligned with increases in the supply and diversity of available vaccines. Low- and middle-income countries (LMICs), however, were less likely to recommend vaccination in pregnancy, or indeed to have developed any policy on the use of COVID-19 vaccines in pregnancy [7]. Despite the global availability of COVID-19 vaccines and the growing body of evidence and expert recommendations to support COVID-19 vaccine use in pregnancy [8–10], some countries retained highly restrictive policies as late as January 2023. In this paper, we aimed to understand and characterize those countries and territories that maintained restrictive policies over time, and to investigate the previously unexplored role of gender inequity in helping to predict policy positions on COVID-19 vaccine administration in pregnancy.

Decades of research have demonstrated that gender inequality and harmful gender norms negatively impact health [11]. Increasingly, scholars and advocates are challenging the role of health systems in perpetuating gender inequality for clients as well as health workers [12, 13]. A core argument is that health systems, being social organizations, replicate and reinforce the harmful gender norms and ideologies of the communities and societies they are situated in through policies, practices, and their own norm-setting influence [12, 14].

Vaccine research and introduction may similarly be influenced by gender bias, including in the context of vaccination during pregnancy. Despite existing frameworks and guidance on the ethical inclusion of pregnant people in vaccine trials for emerging pathogens [15, 16], they were excluded from phase III COVID-19 vaccine trials. Many have criticized the exclusion of pregnant people from clinical research —including research on COVID-19 vaccines and treatments—as both unjust and inimical to public health [17, 18]. Some have argued that this systematic exclusion is rooted in patriarchal beliefs about pregnant women as “vessels” of fetuses, undermining pregnant people’s own ability to make informed decisions about participating in clinical trials, and denying them fair benefit from public investment in biomedical research [14]. While there is growing consensus that pregnant people should have fair opportunity to participate in research that poses the potential for direct benefit and no undue risks to the pregnant person or fetus, the evidence gap for the prevention and treatment of illnesses in pregnancy is far from comparable to that of other adults [19, 20], and consequences persist. Most countries now recommend COVID-19 vaccines in pregnancy, but the delays in access and continued exclusion of pregnant persons in some countries undoubtedly contributed to harms that could have been prevented. In line with this scholarship, our team hypothesized that gender-related ideologies may have influenced countries that implemented and subsequently maintained restrictive policies on COVID-19 vaccination for pregnant people. The authors use the term "pregnant people" to recognize the diversity of those who experience pregnancy. However, because the lived, social experience of pregnancy is shaped by ideologies about women and women's bodies–regardless of an individual's own identity–there are times it is appropriate to refer to women.

National-level composite measures of gender equity/equality have been associated with maternal [21, 22], child [23], and neonatal mortality [24]; and with childhood immunization coverage [25, 26], but to the authors’ knowledge, they have not been studied in the context of maternal immunization policies. We used the World Economic Forum’s Global Gender Gap Index (GGGI) [27], a broad measure of gender equity, to explore associations between a country’s policies on COVID-19 immunization in pregnancy and its relative level of gender equity, while considering country income-level and vaccine availability over the course of the pandemic.

## Methods

### Data collection and sources

From May 2021 to January 2023, our data collection team screened national public health authority web pages for current policies on COVID-19 vaccine eligibility in 224 countries and territories every three weeks. Information on vaccine eligibility for specific groups and guidance on the use of vaccines in pregnant populations were extracted from eligible sources, including national vaccine deployment plans, fact sheets, frequently asked questions, press releases, official social media posts, and screening checklists or consent forms. In a few cases, non-governmental media sources were used when the information could be verified by a secondary source, usually an in-country professional.

### Policy categorization

Policies were analyzed and categorized into one of 6 types to capture variation in national recommendations for use of COVID-19 vaccines in pregnancy. These include: 1) Recommended for some or all: some or all pregnant people should receive a vaccine; 2) Permitted: all pregnant people can receive, may receive, or can choose to receive a vaccine; 3) Permitted with qualifications: only certain groups of pregnant people, e.g., pregnant health workers, pregnant people with underlying conditions, may, or can choose to receive a vaccine; 4) Not recommended but with exceptions: pregnant people should not receive a vaccine, with certain exceptions; 5) Not recommended: pregnant people should not receive a vaccine or a vaccine is contraindicated; and 6) No position found: eligibility information for other groups, such as adults with comorbidities, could be found, but positions regarding pregnancy were missing or no position was clearly established, e.g., ‘if pregnant, talk to your doctor.’ It is worth noting that ‘no position’ is different from ‘missing data.’ A country was classified as having no position on COVID-19 vaccination during pregnancy when some information on COVID-19 vaccine eligibility for other groups was found for that country, whereas missing was assigned when no COVID-19 vaccine administration and eligibility information was found. For each country, every resource containing information on vaccine administration during pregnancy was included and coded, including different positions based on vaccine product or policymaking body and changing positions over time. To assign a country-level policy code at a given time, the most permissive position of all updated policies for that country was selected. For example, if a country’s position on an mRNA vaccine product was “permitted” and their position on a non-mRNA vaccine product was “not recommended”, then the overall country code for the use of a COVID-19 vaccine in pregnancy was considered “permitted”. Our data, along with detailed information on our data collection procedures, can be found at https://www.comitglobal.org/.

### Outcomes

For this analysis, these policy categories were used to develop two outcomes of interest. The primary outcome was defined as the proportion of countries with restrictive policies at three timepoints in June 2021, October 2021, and March 2022. June 1st, 2021, represented the earliest time point where we had comprehensive policy determination data from 224 countries and territories. October 15th, 2021, was selected as the second time point, as the risks of COVID-19 during pregnancy were by then well documented [1, 28–33], and the evidence on the safety of mRNA COVID-19 vaccines during pregnancy was strong [34–37]. March 1st, 2022, was selected to capture a time point after the World Health Organization (WHO) provided unambiguous guidance recommending use of COVID-19 vaccines in pregnancy [9], and the global supply of COVID-19 vaccines was not constrained [38]. Policy categories 4 and 5 were together labeled “Restrictive”, categories 1, 2 and 3 were together labeled “Permissive”, and “no position” was considered missing data. It is possible that having no position on the use of COVID-19 vaccine administration in pregnancy reflected an assumption that these vaccines would not be used during pregnancy. However, to take the most conservative approach to this analysis, we intentionally did not include countries without a position on COVID-19 vaccine administration in pregnancy.

The second outcome of interest was defined as a change in policy from restrictive or no position in June 2021 to permissive in October 2021 as this was the time period in which the largest number of countries switched policies. For this outcome, we did include countries with no position as of June 2021, as they had existing policies on COVID-19 vaccine eligibility, but not for pregnant people, so it was worthwhile to consider what factors may have influenced those countries to switch from no position to permissive. However, countries with no information whatsoever on COVID-19 vaccine administration policies were always excluded from analyses, as there are likely more important factors at play, such as overall health system functioning.

### Predictors

We explored three potential country-level predictors for the two outcomes: country/territory income level, availability of mRNA vaccines, and gender equity score. The choice of income level and availability of mRNA vaccines was based upon our previous work that identified higher income and availability of mRNA vaccines as predictive of more permissive policies [7]. Country income level was defined according to the World Bank as of 2021. Data on vaccine platform and supply in each country or territory over time was extracted from the Our World in Data database [39]. A variable was generated for each timepoint to capture whether a country did or did not have any mRNA vaccine supply at that timepoint. In the early phase of COVID-19 vaccine rollouts, some countries including Kenya, France, Brazil, and others, did not recommend the use of viral vector vaccines in pregnancy, but did recommend them for older adults and those with comorbidities, whereas mRNA vaccines were specifically recommended for pregnant people. Similarly, on June 15th, 2021, the World Health Organization issued guidance on the use of the Pfizer/BioNTech and Moderna mRNA vaccines in pregnancy, explicitly permitting their use for all pregnant people while at the same time, permitting with qualifications the Johnson & Johnson and Oxford/Astrazeneca vaccines in pregnancy, establishing at the time a preference for mRNA vaccine use in pregnancy where different platforms were available [40–43]. Additionally, the WHO SAGE guidance was updated to include pregnant people alongside those at elevated risk of severe COVID-19 illness in vaccine prioritization [44]. This observed preference for mRNA vaccines in pregnancy along with prioritization of pregnant people for vaccination was the basis for studying mRNA availability as a binary variable, as decision makers might choose to amend their policy on vaccine use in pregnancy once mRNA COVID-19 vaccines were available in their country. The Global Gender Gap Index score (GGGI) from the World Economic Forum’s 2021 Global Gender Gap report was used as the indicator for gender equity [27]. The GGGI score is a composite of 14 indicators covering economic opportunities, education, health and political leadership; with scores closer to zero indicating a high gender gap (inequity) and scores closer to 1 indicating no gender gap (equity). In 2021, 156 countries were given a score, ranging from 0.44 to 0.89.

### Statistical analysis

We explored the proportions of restrictive and permissive policy positions by income level, availability of mRNA vaccines, and by GGGI score at the three timepoints. Simple logistic regression models were conducted to assess how each of the three predictors independently influenced the odds of a restrictive policy at each timepoint, followed by multivariable logistic regression models to evaluate whether income level and mRNA vaccine availability modified the relationship between gender equity and restrictive policy at each timepoint. We conducted a sensitivity analysis to explore whether statistical results remained robust when outliers with very low GGGI scores were excluded. We next used simple and multivariable logistic regression models to explore whether the odds of a change in policy from restrictive/no position to permissive from June 1st to October 15th, 2021, was influenced by any of the three predictors of interest. This analysis was restricted to 120 countries/territories that had a restrictive policy or no position in June 2021. 89 countries that already had permissive positions in June and 15 countries where no data on vaccine eligibility more broadly could be found were excluded. Statistical analyses were conducted in Stata, version 15.

The funders of the study had no role in study design, data collection, data analysis, data interpretation, or writing of the report. Patients and the public were not involved in the design or conduct of this study. However, data that informed these findings are available to the public through our website, www.comitglobal.org.

## Results

### Data availability

**Fig. 1:**
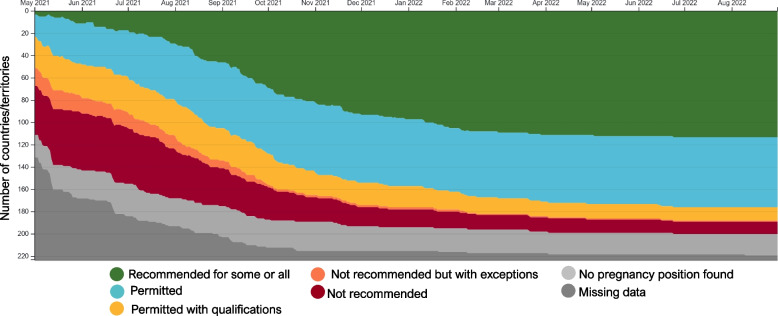
Trends in policies for the use of COVID-19 vaccines in pregnancy, May 2021 to August 2022

Data on income level and platform availability were available for all 224 countries and territories. The availability of maternal immunization policies increased over time as countries established policies over the course of their vaccination campaigns. Figure 1 illustrates the trend of COMIT policy positions over time, with high variability and frequent policy absence in May 2021, followed by a decline in restrictive policies and an increase in permissive ones through March 2022, and then a general plateau in policy position changes. As of January 5th, 2023, when data collection was completed, 10 countries still maintained restrictive policies for the use of COVID-19 vaccines in pregnancy: Afghanistan, China, Côte d’Ivoire, Djibouti, Jordan, Sierra Leone, Timor-Leste, Turkmenistan, United Arab Emirates, and Uzbekistan. For another 20 countries and territories, 16 of them in sub-Saharan Africa, we were unable to find policy positions over the duration of data collection and they were therefore excluded from the analysis.

For the binary outcome of permissive versus restrictive policy at three timepoints, the proportion of countries and territories with a permissive policy position increased over time. In June 2021, 58.2% had a permissive policy position, followed by 86.7% in October 2021, and 93.4% in March 2022.

**Fig. 2:**
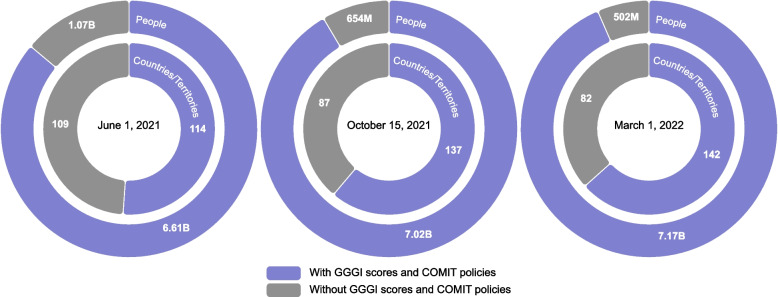
Availability of country level COMIT policy data and GGGI scores, from June 2021 to March 2022. Legend: COMIT: COVID-19 Maternal Immunization Tracker; GGGI: Global Gender Gap Index. World population data were obtained from the World Bank, 2019 estimates

GGGI scores were available for 156 countries and territories. Complete data that included vaccine policies, income levels, mRNA vaccine availability, and GGGI scores were available for 114 countries/territories in June 2021, 137 in October 2021, and 142 in March 2022. The 142 countries with complete data on March 1st, 2022, represented roughly 7.2 billion people, or about 93% of the world population (Fig. 2).

### Gender equity and restrictive policies

**Fig. 3:**
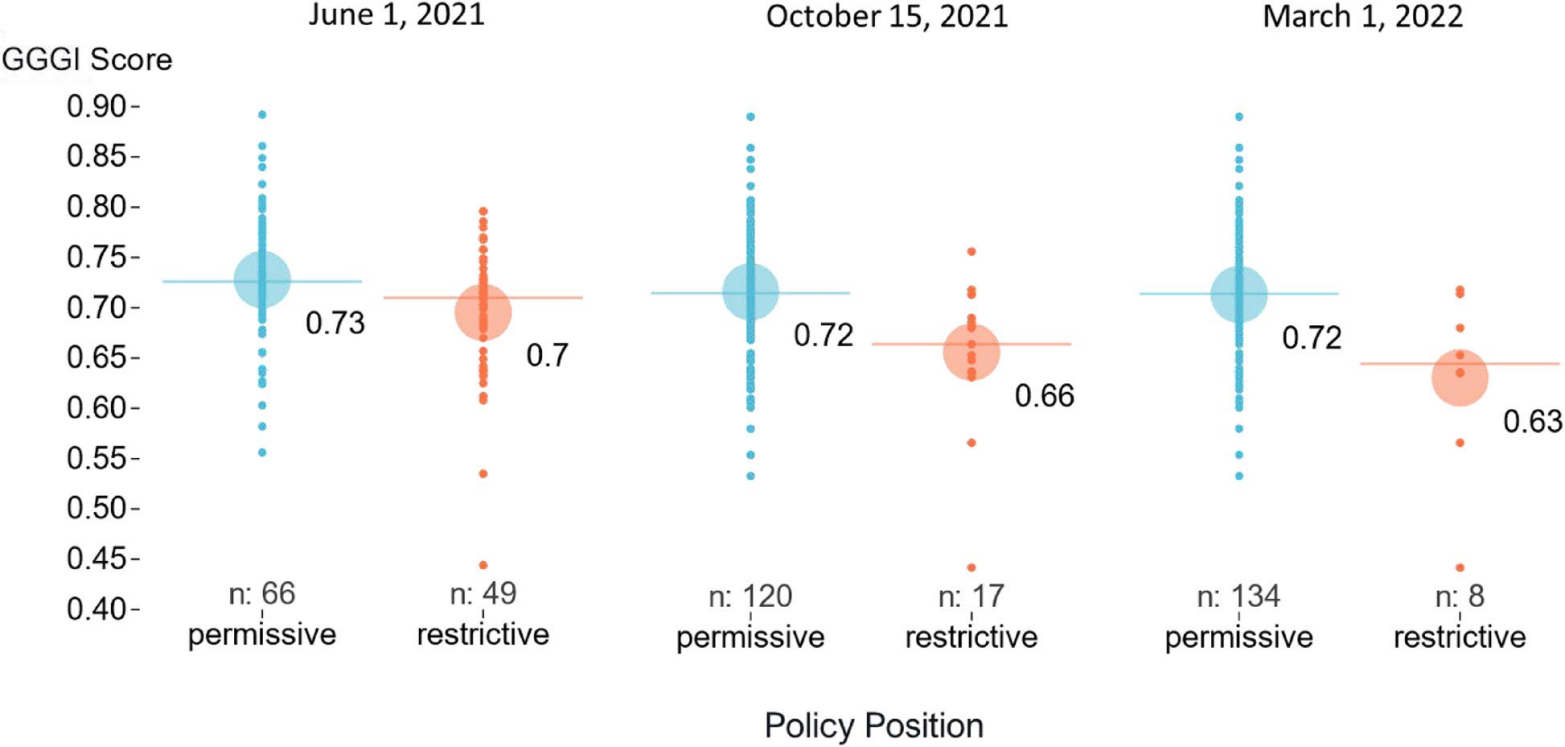
Mean GGGI score by COVID-19 vaccine policy position for use in pregnancy, from June 2021 to March 2022. Legend: Blue represents a permissive policy position and orange represents a restrictive policy position. The small circles represent individual country scores, the horizontal lines represent the group medians, and the large circles and adjacent numbers represent the group means. GGGI: Global Gender Gap Index

Figure 3 illustrates that the means and distributions of GGGI scores were lower for countries with a restrictive policy compared to those with a permissive policy. While the mean GGGI score for countries with a permissive policy stayed constant across the three timepoints, between 0.72 and 0.73, the mean GGGI score among restrictive policy countries declined over time, from 0.70 in June 2021 to 0.66 in October 2021 and 0.63 in March 2022, creating a wider gap in gender equity scores between countries with a permissive stance on the use of COVID-19 vaccines in pregnancy versus those with a restrictive stance (Supplementary Table 1). The simple regression models revealed that better gender equity scores were associated with significantly reduced odds of a restrictive policy at all three timepoints (Table 1). The reduction in the odds increased over time, from 0.92 (95%CI: 0.87, 0.98) in June 2021 to 0.84 (95%CI: 0.75, 0.94) in March 2022, indicating that gender equity had a stronger association with policy at the later time points. These results remained significant when Afghanistan, the country with the lowest GGGI score in 2021 (0.44), was excluded (Supplementary Table 2).

**Table 1:** Odds of a restrictive policy position by independent variables at three timepoints, unadjusted

	6/1/2021				10/15/2021				3/1/2022			
	n	cOR	95%CI	*p*-val	n	cOR	95%CI	*p*-val	n	cOR	95%CI	*p*-val
GGGI	114	0.92	0.87, 0.98	0.013	137	0.87	0.80, 0.95	0.001	142	0.84	0.75, 0.94	0.003
mRNA vaccine	146	0.34	0.17, 0.67	0.002	188	0.28	0.12, 0.67	0.004	198	0.79	0.23, 2.68	0.703
Income level												
Low	4	2.80	0.37, 21.22	0.319	11	17.29	3.66, 81.80	0.000	16	6.54	1.19, 35.91	0.031
Low middle	26	5.29	2.03, 13.75	0.001	38	6.44	1.84, 22.51	0.004	41	3.06	0.65, 14.37	0.156
Upper middle	40	3.42	1.53, 7.66	0.003	52	3.23	0.90, 11.62	0.073	53	1.70	0.33, 8.75	0.525
High	76	1.00	··		87	1.00	··		88	1.00	··	

*cOR* Crude odds ratio, *GGGI* Global Gender Gap Index

### Income-level and restrictive policies

Analysis of the frequency of policy positions by income level revealed that while the proportion of restrictive policies decreased over time at all income levels, low and lower-middle income countries had a greater proportion of restrictive policy positions over time compared to upper-middle- and high-income countries (Supplementary Table 3). The simple logistic regression models revealed that the odds of a restrictive policy in upper-middle and low-middle income countries as compared to high-income countries decreased over time. However, the odds ratio of a restrictive policy in non-high-income countries at any timepoint was large, with the upper bounds of the confidence intervals far from the null, indicating a greater likelihood of restrictive policies in non-high-income countries across the study period. In low-middle income countries, the odds of a restrictive policy in June 2021 was 5.29 (95%CI: 2.03, 13.75) compared to 3.06 (95%CI: 0.65, 14.37) in March 2022. However, the odds of restrictive policy in low-income countries when compared to high-income countries persisted through March 2022 (crude odds ratio (cOR): 6.54, 95%CI: 1.19, 35.91) (Table 1). We also note a non-significant odds ratio at the June 2021 time point for low-income countries, but this can be explained by the small sample size: only 4 low-income countries had a COVID-19 maternal immunization policy at that time.

### mRNA vaccine availability and restrictive policies

Countries in which mRNA vaccines were available had a smaller proportion of restrictive policies compared to those without mRNA vaccines (Supplementary Table 4). However, this difference in proportions was strongest in June and October, whereas in March, countries without access to mRNA vaccines were also adopting more permissive policies, and the difference in proportions was diminished. The logistic regression model confirmed that countries with mRNA vaccines had lower odds of a restrictive policy in June (cOR: 0.34, 95%CI: 0.17, 0.67) and October (cOR: 0.28, 95%CI: 0.12, 0.67), but this effect was no longer significant in March of 2022 (cOR: 0.79, 95%CI: 0.23, 2.68) (Table 1).

### Gender equity and restrictive policies, adjusted for income level and vaccine availability

After adjusting for income level and mRNA vaccine availability, higher GGGI scores reduced the relative odds of a restrictive COVID-19 vaccine policy by 10% (adjusted odds ratio (aOR): 0.90, 95CI: 0.81, 0.99) in October 2021 and 14% (aOR: 0.86, 95%CI: 0.76, 0.97) in March 2022, as seen in Table 2. However, at the earliest time point, GGGI score did not influence the odds of a restrictive policy position when adjusted for income level and mRNA vaccine availability. In the sensitivity analysis where Afghanistan was excluded, higher GGGI scores did not reduce the odds of a restrictive COVID-19 vaccine policy in October 2021 (aOR: 0.91, 95%CI: 0.82, 1.00), but did reduce the odds of a restrictive policy in March 2022 (aOR: 0.87, 95%CI: 0.76, 0.99) (Supplementary Table 2).

**Table 2:** Odds of a restrictive policy position by GGGI score at three time points, adjusted for country income level and mRNA vaccine availability

	6/1/2021				10/15/2021				3/1/2022			
	n	aOR^a^	95%CI	*p*-val	n	aOR	95%CI	*p*-val	n	aOR	95%CI	*p*-val
GGGI	114	0.95	0.89, 1.02	0.147	137	0.90	0.81, 0.99	0.029	142	0.86	0.76, 0.97	0.016

*aOR* Adjusted odds ratio, *GGGI* Global Gender Gap Index

^a^Multiple logistic regression adjusted for country income level and mRNA vaccine availability

Table 3 details the GGGI scores, income-level, and policy positions for adults over the age of 18 in countries that still maintained a restrictive policy on COVID-19 vaccine administration in pregnancy as of March 2022.

**Table 3:** Comparison of policy positions for all adults versus pregnant people as of March 2022 for countries with a restrictive policy position in pregnancy

Country	Policy for all adults	Policy for pregnant people	GGGI score	Income Status
Afghanistan	All people above the age of 18 were permitted to receive the vaccine, from April 12, 2021. Pregnant women were explicitly excluded in MoPH announcement [45]	Not recommended	0.444	Low income
China	All people above the age of 18 were permitted to receive the vaccine from late March 2021. Pregnant women were still considered "contraindicated" at this date, from China CDC documents [46]	Not recommended	0.682	Upper middle income
Côte d'Ivoire	As of March 29, 2021, Cote d'Ivoire opened up their vaccination campaigns to any adult over the age of 18 [47]	Not recommended with exceptions	0.637	Lower middle income
Jordan	Jordan invited all residents of Jordan over the age of 18 to be vaccinated for COVID-19 when they initiated their vaccine campaign in January 2021[48]. Pregnant women were explicitly excluded from vaccination [49]	Not recommended	0.638	Upper middle income
Sierra Leone	In Sierra Leone's COVID-19 Strategic Plan, originally published June 2021 and updated September 2022, they list that Phase 3 prioritization for vaccination includes 'those aged 18–59, without comorbidities, not including pregnant women' [50]	Not recommended	0.655	Low income
Syria	As of early September 2021, Syria was conducting a vaccination campaign targeting all citizens over the age of 18. Pregnancy and lactation were explicitly called out as 'conditions that prevent vaccination' [51]	Not recommended	0.568	Low income
Timor-Leste	While it's not clear when Timor-Leste started vaccinating all adults over the age of 18, there is evidence that by August 2021, over half the population over 18 had received at least one dose, indicating all adults were eligible to receive the vaccine before August 2021 [52]	Not recommended	0.72	Lower middle income
United Arab Emirates	As of March 22, 2021, the UAE was administering vaccines to adults 18 years of age and older. Pregnant women continued to be excluded/exempted from vaccination in available policies [53]	Not recommended	0.716	High income

### Predictors of a change in policy

Of the 120 countries/territories that had a restrictive policy or no position on the use of COVID-19 vaccines in pregnancy on June 1st, 2021, 70 (58%) switched to permissive and 50 (42%) remained restrictive or had no position by October 15th, 2021. Table 4 demonstrates that in the simple regression models, a higher GGGI score and the availability of mRNA vaccines as of October increased the odds of a switch to a permissive policy (GGGI cOR: 1.15, 95%CI: 1.05, 1.27; mRNA vaccine cOR: 6.21, 95%CI: 2.77, 13.94), whereas a low or low middle income classification decreased the odds of a switch (Low income cOR: 0.04, 95%CI: 0.01, 0.19; Low-middle income cOR: 0.20, 95%CI: 0.06, 0.63). After adjusting for income level and mRNA vaccine availability, higher GGGI scores were associated with a 12% increase (aOR: 1.12, 95%CI: 1.00, 1.24) in the relative odds of a switch from restrictive/no position to a permissive policy on the use of COVID-19 vaccines in pregnancy.

**Table 4:** Odds of a policy change from restrictive or no position in June to permissive in October, by GGGI, mRNA vaccine availability, and country income-level

	n	cOR	95%CI	*p*-val	aOR	95%CI	*p*-val
GGGI	81	1.15	1.05, 1.27	0.003	1.12	1.00, 1.24	0.043
mRNA vaccine	120	6.21	2.77, 13.94	0.000	3.11	0.92, 10.49	0.068
Income level							
Low	19	0.04	0.01, 0.19	0.000	0.08	0.01, 0.88	0.039
Low middle	35	0.20	0.06, 0.63	0.006	0.43	0.07, 2.73	0.372
Upper middle	37	0.49	0.15, 1.62	0.245	1.20	0.17, 8.55	0.858
High	29	1.00	··		1.00	··	

*cOR* Crude odds ratio, *aOR* Adjusted odds ratio, *GGGI* Global Gender Gap Index

^1^Multiple logistic regression adjusted for the two variables not indicated by the independent variable: GGGI, country income level, and/or mRNA vaccine availability

## Discussion

By March 2022, more than 90% of countries with available policies had permissive positions regarding the use of COVID-19 vaccines in pregnancy. We found that as evidence about COVID-19 disease in pregnancy and vaccine supply increased, gender equity became an increasingly important policy predictor, supporting our hypothesis that lingering restrictive policies may be driven in part by harmful gender norms and attitudes, along with persistent inequities reflected in country economic status.

The evidence presented here is specific to COVID-19 vaccine administration policy, not implementation. For this reason, we did not explore factors that may be related to the feasibility of expanding COVID-19 vaccination to pregnant people, such as measures of routine vaccine coverage or access to prenatal care. Moreover, the countries shown in Table 3, which had specific policy restrictions related to the use of these vaccines in pregnancy even as they encouraged vaccine use in other populations, represented a range of income levels and health systems of various strengths. While implementation considerations are relevant to policy formulation, they do not justify denying COVID-19 vaccine to a high- risk group, as a restrictive policy would do. In fact, all the countries and territories included in this analysis had issued policy positions on vaccine administration and eligibility for other adult populations, for whom there may also have been implementation challenges, suggesting any of these countries could have reasonably taken or changed their position on vaccination in pregnancy. It is plausible that initial delays in permitting COVID-19 vaccine administration in pregnancy may have been related to the lack of evidence on safety for non-mRNA vaccine platforms; countries without the cold chain to provide mRNA vaccines may have delayed taking or switching positions. However, well before March 2022, there was sufficient evidence on the safety of other COVID-19 vaccine platform types in pregnancy and the WHO SAGE guidance explicitly recommended all pregnant people have access to COVID-19 vaccines and did not restrict this recommendation to only mRNA vaccines [9]. Thus, at least according to the WHO, the absence of advanced cold chain capacity should not have been a barrier to permissive policies in pregnancy.

This study contributes to the body of evidence that health policies may be influenced by harmful gender norms and ideologies, and that health policies themselves can serve to perpetuate inequity in denying adult pregnant persons the right to make informed decisions about their bodies. It should go without saying that denying vaccines to an at-risk group is unethical and results in preventable harms. As research and programming on health systems strengthening evolves, it is critical to interrogate the assumptions and values on which policies, practices, and structures are based. Policies and programs that actively promote greater gender equity will likely result in better, more just, and more sustainable health outcomes.

It is likely that gendered ideologies contributed to a factor that affected vaccine policy from the beginning- the absence of pregnancy-specific data when vaccines were first given emergency use authorization. Exclusion of pregnant people from participation in clinical trials, as well as the failure to otherwise generate timely evidence in pregnancy, contributed to delays in policy makers recommending vaccines for pregnant people, which then resulted in delays in pregnant people’s access to a life-saving intervention. The absence of relevant data likely had a lasting impact on uptake of the vaccine in pregnancy by further undermining trust in the vaccine by pregnant people, healthcare providers, and the public. However, our findings suggest that before and after evidence on the increased risk of COVID-19 in pregnancy and on the vaccine’s safety in pregnancy were available, gender inequity was associated with inequitable, pregnancy-specific vaccine policy.

Policies that permit pregnant persons to receive COVID-19 vaccines represent the first step towards access, but gender inequality poses further barriers to COVID-19 vaccine uptake beyond policy. Several multi-country meta-analyses found women were significantly more likely than men to express vaccine hesitancy [54–56], and actual vaccine uptake by gender has varied across contexts [57–61]. Further, pregnant and breastfeeding populations globally were found to have higher vaccine hesitancy and lower vaccine uptake than the general population [57, 62, 63]. Studies across different settings suggest COVID-19 vaccine hesitancy in pregnancy and lactation stems from fears about the safety and side effects of the vaccine [63–65]. Additionally, studies from Ghana and India have documented gender-related barriers to women’s vaccine access such as childcare, informal work duties and lower digital literacy, access, and usage [66]. To optimize the benefits of vaccines to women and pregnant people, findings from our work and these previous studies highlight the importance of attending to gender equity along the continuum from vaccine research and development to authorization, policy, and program implementation.

Several countries and territories did not contribute data, limiting the generalizability of these findings. Countries with missing information about pregnancy policies, GGGI scores, or both were predominantly low income. Afghanistan was observed to be a possible outlier for having both a very low GGGI score (0.444) and having a policy on the use of COVID-19 vaccines in pregnancy. Some other countries with low GGGI scores, such as Yemen (0.492), DR Congo (0.576), Mali (0.591), and Chad (0.593) did not have available policies on COVID-19 vaccine use in pregnancy; it is therefore unknown how their inclusion would have influenced these findings. While these exclusions potentially limit the generalizability of our findings, we felt it was important to exclude countries with missing policies from the analysis of countries with restrictive policies, as we could not determine whether any relevant policies, restrictive or otherwise, were in place. Several small high-income territories had vaccination policies, but not GGGI scores; however, the countries and territories that did contribute data represented 93% of the world population in March 2022. Even when COVID-19 vaccines were widely available and evidence on the safety and benefit of COVID-19 vaccines in pregnancy was established, countries with lower gender equity scores continued to restrict access to lifesaving vaccines for pregnant people. This paper is the first, to the authors’ knowledge, to assess the relationship between national gender equity measures and maternal immunization policies at a global scale.

## Conclusions

There is growing consensus that pregnant people should never again be left behind in access to medical countermeasures (MCM) for public health emergencies. Efforts are underway to develop model protocols and oversight guidance for the timely and ethical inclusion of pregnant people in MCM research and development. Our findings suggest that these efforts, while welcome and necessary, will not be sufficient to ensure pregnant people are treated fairly in public health policies and access. It will be important that vaccines produced through a research and development process that includes pregnant people are also available to pregnant people throughout the world. Moreover, as is the case with gender inequities in health and well-being more broadly, our findings suggest that pandemic vaccine policies also may be rooted in deeply embedded cultural biases and unfair patterns of power that need to be addressed if fairness for pregnant people in pandemic response is to be secured.

Vaccine policy is almost certainly not an outlier as a policy informed by harmful gender ideologies. Health systems research must continue to study the mechanisms by which policies, programs, and structures are informed by cultural values, and the impact of these values on the health and rights of various segments of the population. Health systems must seek to deliberately interrupt harmful norms and promote greater equity and health for all.

## Supplementary Information

Supplementary Material 1.

## Data Availability

The datasets generated and analysed in the current study are available at www.comitglobal.org.

## Abbreviations

COMIT: COVID-19 Maternal Immunization Tracker
LMICs: Low- and Middle-Income Countries
WHO: World Health Organisation
GGGI: Global Gender Gap Index
MCM: Medical Countermeasures

## References

[1] Allotey J, Stallings E, Bonet M, Yap M, Chatterjee S, Kew T, et al. Clinical manifestations, risk factors, and maternal and perinatal outcomes of coronavirus disease 2019 in pregnancy: living systematic review and meta-analysis. BMJ. 2020;1(370):m3320. 10.1136/bmj.m3320 PMC7459193 32873575

[2] McClymont E, Albert AY, Alton GD, Boucoiran I, Castillo E, Fell DB, et al. Association of SARS-CoV-2 infection during pregnancy with maternal and perinatal outcomes. JAMA. 2022;327(20):1983–91. 35499852 10.1001/jama.2022.5906 PMC9062768

[3] Lokken EM, Huebner EM, Taylor GG, Hendrickson S, Vanderhoeven J, Kachikis A, et al. Disease severity, pregnancy outcomes, and maternal deaths among pregnant patients with severe acute respiratory syndrome coronavirus 2 infection in Washington State. Am J Obstet Gynecol. 2021;225(1):77.e1-77.e14. 33515516 10.1016/j.ajog.2020.12.1221 PMC7838012

[4] Sadarangani M, Soe P, Shulha HP, Valiquette L, Vanderkooi OG, Kellner JD, et al. Safety of COVID-19 vaccines in pregnancy: a Canadian National Vaccine Safety (CANVAS) network cohort study. Lancet Infect Dis. 2022;22(11):1553–64. Available from: https://www.sciencedirect.com/science/article/pii/S1473309922004261. Cited 2022 Aug 17. 35964614 10.1016/S1473-3099(22)00426-1 PMC9371587

[5] Morgan JA, Biggio JRJ, Martin JK, Mussarat N, Chawla HK, Puri P, et al. Maternal outcomes after severe acute respiratory syndrome coronavirus 2 (SARS-CoV-2) infection in vaccinated compared with unvaccinated pregnant patients. Obstet Gynecol. 2022;139(1):107–9. 34644272 10.1097/AOG.0000000000004621

[6] Halasa NB. Effectiveness of Maternal Vaccination with mRNA COVID-19 Vaccine During Pregnancy Against COVID-19–Associated Hospitalization in Infants Aged 6 Months — 17 States, July 2021–January 2022. MMWR Morb Mortal Wkly Rep. 2022;71. Available from: https://www.cdc.gov/mmwr/volumes/71/wr/mm7107e3.htm. Cited 2022 May 27. 10.15585/mmwr.mm7107e3 PMC8853480 35176002

[7] Zavala E, Krubiner CB, Jaffe EF, Nicklin A, Gur-Arie R, Wonodi C, et al. Global disparities in public health guidance for the use of COVID-19 vaccines in pregnancy. BMJ Glob Health. 2022;7(2):e007730. 35210309 10.1136/bmjgh-2021-007730 PMC8882664

[8] American College of Obstetrics. COVID-19 Vaccination Considerations for Obstetric–Gynecologic Care. 2020. Available from: https://www.acog.org/clinical/clinical-guidance/practice-advisory/articles/2020/12/covid-19-vaccination-considerations-for-obstetric-gynecologic-care. Cited 2023 Sep 20.

[9] World Health Organization. Questions and Answers: COVID-19 vaccines and pregnancy. 2022. Available from: https://www.who.int/publications-detail-redirect/WHO-2019-nCoV-FAQ-Pregnancy-Vaccines-2022.1. Cited 2023 Sep 20.

[10] Royal College of Obstetricians & Gynaecologists. COVID-19 vaccines, pregnancy and breastfeeding FAQs. RCOG. 2022. Available from: https://www.rcog.org.uk/guidance/coronavirus-covid-19-pregnancy-and-women-s-health/vaccination/covid-19-vaccines-pregnancy-and-breastfeeding-faqs/. Cited 2023 Sep 20.

[11] Heise L, Greene ME, Opper N, Stavropoulou M, Harper C, Nascimento M, et al. Gender inequality and restrictive gender norms: framing the challenges to health. Lancet Lond Engl. 2019;393(10189):2440–54. 10.1016/S0140-6736(19)30652-X 31155275

[12] Hay K, McDougal L, Percival V, Henry S, Klugman J, Wurie H, et al. Disrupting gender norms in health systems: making the case for change. Lancet Lond Engl. 2019Jun 22;393(10190):2535–49. 10.1016/S0140-6736(19)30648-8 PMC7233290 31155270

[13] Women in Global Health. Women in Global Health. Policy Brief: The State of Women and Leadership in Global Health: The XX Paradox. 2023. Available from: https://womeningh.org/wp-content/uploads/2023/03/WGH-Policy-Report-2023-2-1.pdf.

[14] Ambrogi I, Brito L, Griner A, Bull S. Gender inequity and COVID-19 vaccination policies for pregnant women in the Americas. Wellcome Open Res Open Access Publ Platf. 2023. Available from: https://wellcomeopenresearch.org/articles/8-121. Cited 2023 Dec 21.

[15] Lyerly AD, Beigi R, Bekker LG, Chi BH, Cohn SE, Diallo DD, et al. Ending the evidence gap for pregnancy, HIV and co-infections: ethics guidance from the PHASES project. J Int AIDS Soc. 2021;24(12):e25846. 34910846 10.1002/jia2.25846 PMC8673925

[16] Krubiner CB, Faden RR, Karron RA, Little MO, Lyerly AD, Abramson JS, et al. Pregnant women & vaccines against emerging epidemic threats: Ethics guidance for preparedness, research, and response. Vaccine. 2021;39(1):85–120. 31060949 10.1016/j.vaccine.2019.01.011 PMC7735377

[17] Waitt C, Astill D, Zavala E, Karron RA, Faden RR, Stratton P, et al. Clinical trials and pregnancy. Commun Med. 2022;2(1):1–5. 36299562 10.1038/s43856-022-00198-1 PMC9593973

[18] Van Spall HGC. Exclusion of pregnant and lactating women from COVID-19 vaccine trials: a missed opportunity. Eur Heart J. 2021;42(28):2724–6. 33686419 10.1093/eurheartj/ehab103 PMC7989536

[19] Task force on research specific to pregnant women and lactating women. PRGLAC Report to the Secretary of Health and Human Services and Congress. 2018. Available from: https://www.nichd.nih.gov/sites/default/files/2018-09/PRGLAC_Report.pdf.

[20] Ren Z, Bremer AA, Pawlyk AC. Drug development research in pregnant and lactating women. Am J Obstet Gynecol. 2021;225(1):33–42. 33887238 10.1016/j.ajog.2021.04.227

[21] Choe SA, Cho SI, Kim H. Gender gap matters in maternal mortality in low and lower-middle-income countries: A study of the global Gender Gap Index. Glob Public Health. 2017;12(9):1065–76. 27021475 10.1080/17441692.2016.1162318

[22] Bagade T, Chojenta C, Harris M, Oldmeadow C, Loxton D. The human right to safely give birth: data from 193 countries show that gender equality does affect maternal mortality. BMC Pregnancy Childbirth. 2022;22(1):874. 36424537 10.1186/s12884-022-05225-6 PMC9685845

[23] Bagade T, Chojenta C, Harris M, Oldmeadow C, Loxton D. A women’s rights-based approach to reducing child mortality: data from 193 countries show that gender equality does affect under-five child mortality. Matern Child Health J. 2022;26(6):1292–304. 34982333 10.1007/s10995-021-03315-z

[24] Abdollahpour S, Heidarian Miri H, KhademolKhamse F, Khadivzadeh T. The relationship between global gender equality with maternal and neonatal health indicators: an ecological study. J Matern-Fetal Neonatal Med Off J Eur Assoc Perinat Med Fed Asia Ocean Perinat Soc Int Soc Perinat Obstet. 2022Mar;35(6):1093–9. 10.1080/14767058.2020.1743655 32290738

[25] Johns NE, Santos TM, Arroyave L, Cata-Preta BO, Heidari S, Kirkby K, et al. Gender-related inequality in childhood immunization coverage: a cross-sectional analysis of dtp3 coverage and zero-dose DTP prevalence in 52 countries using the SWPER global index. Vaccines. 2022;10(7):988. 35891152 10.3390/vaccines10070988 PMC9315814

[26] Johns NE, Kirkby K, Goodman TS, Heidari S, Munro J, Shendale S, et al. Subnational gender inequality and childhood immunization: an ecological analysis of the subnational gender development index and DTP coverage outcomes across 57 countries. Vaccines. 2022;10(11):1951. 36423046 10.3390/vaccines10111951 PMC9698767

[27] World Economic Forum. Global Gender Gap Report 2021. 2021. Available from: https://www.weforum.org/reports/global-gender-gap-report-2021/. Cited 2023 Sep 20.

[28] Metz TD, Collier C, Hollier LM. Maternal Mortality From Coronavirus Disease 2019 (COVID-19) in the United States. Obstet Gynecol. 2020;136(2):313. 32544145 10.1097/AOG.0000000000004024

[29] Adhikari EH, Moreno W, Zofkie AC, MacDonald L, McIntire DD, Collins RRJ, et al. Pregnancy Outcomes Among Women With and Without Severe Acute Respiratory Syndrome Coronavirus 2 Infection. JAMA Netw Open. 2020;3(11):e2029256. 33211113 10.1001/jamanetworkopen.2020.29256 PMC7677755

[30] Woodworth KR, Olsen EO, Neelam V, Lewis EL, Galang RR, Oduyebo T, et al. Birth and infant outcomes following laboratory-confirmed SARS-CoV-2 infection in pregnancy - SET-NET, 16 jurisdictions, March 29-October 14, 2020. MMWR Morb Mortal Wkly Rep. 2020;69(44):1635–40. 33151917 10.15585/mmwr.mm6944e2 PMC7643898

[31] Zambrano LD. Update: Characteristics of Symptomatic Women of Reproductive Age with Laboratory-Confirmed SARS-CoV-2 Infection by Pregnancy Status — United States, January 22–October 3, 2020. MMWR Morb Mortal Wkly Rep. 2020;69. Available from: https://www.cdc.gov/mmwr/volumes/69/wr/mm6944e3.htm. Cited 2022 Jul 20. 10.15585/mmwr.mm6944e3 PMC7643892 33151921

[32] Papageorghiou AT, Deruelle P, Gunier RB, Rauch S, García-May PK, Mhatre M, et al. Preeclampsia and COVID-19: results from the INTERCOVID prospective longitudinal study. Am J Obstet Gynecol. 2021;225(3):289.e1-289.e17. 34187688 10.1016/j.ajog.2021.05.014 PMC8233533

[33] Gurol-Urganci I, Jardine JE, Carroll F, Draycott T, Dunn G, Fremeaux A, et al. Maternal and perinatal outcomes of pregnant women with SARS-CoV-2 infection at the time of birth in England: national cohort study. Am J Obstet Gynecol. 2021;225(5):522.e1-522.e11. 34023315 10.1016/j.ajog.2021.05.016 PMC8135190

[34] Goldshtein I, Nevo D, Steinberg DM, Rotem RS, Gorfine M, Chodick G, et al. Association between BNT162b2 vaccination and incidence of SARS-CoV-2 infection in pregnant women. JAMA. 2021;326(8):728–35. 34251417 10.1001/jama.2021.11035 PMC8276131

[35] Zauche LH, Wallace B, Smoots AN, Olson CK, Oduyebo T, Kim SY, et al. Receipt of mRNA Covid-19 vaccines and risk of spontaneous abortion. N Engl J Med. 2021;385(16):1533–5. 34496196 10.1056/NEJMc2113891 PMC8451181

[36] Shimabukuro TT, Kim SY, Myers TR, Moro PL, Oduyebo T, Panagiotakopoulos L, et al. Preliminary findings of mRNA Covid-19 vaccine safety in pregnant persons. N Engl J Med. 2021;384(24):2273–82. 33882218 10.1056/NEJMoa2104983 PMC8117969

[37] Dagan N, Barda N, Biron-Shental T, Makov-Assif M, Key C, Kohane IS, et al. Effectiveness of the BNT162b2 mRNA COVID-19 vaccine in pregnancy. Nat Med. 2021;27(10):1693–5. 34493859 10.1038/s41591-021-01490-8

[38] Guarascio F, Rigby J. COVID vaccine supply for global programme outstrips demand for first time. Reuters. 2022. Available from: https://www.reuters.com/business/healthcare-pharmaceuticals/covax-vaccine-supply-outstrips-demand-first-time-2022-02-23/. Cited 2022 Jul 20.

[39] Mathieu E, Ritchie H, Ortiz-Ospina E, Roser M, Hasell J, Appel C, et al. A global database of COVID-19 vaccinations. Nat Hum Behav. 2021;5(7):947–53. 33972767 10.1038/s41562-021-01122-8

[40] World Health Organization. Interim recommendations for use of the Pfizer–BioNTech COVID-19 vaccine, BNT162b2, under Emergency Use Listing. Geneva, Switzerland; 2021. Available from: https://www.who.int/publications/i/item/WHO-2019-nCoV-vaccines-SAGE_recommendation-BNT162b2-2021.1. Cited 2025 Feb 3.

[41] World Health Organization. Interim recommendations for use of the Moderna mRNA-1273 vaccine against COVID-19 [Internet]. Geneva, Switzerland; 2021. Available from: https://www.who.int/publications/i/item/WHO-2019-nCoV-vaccines-SAGE-recommendation-mRNA-1273-2021.3. Cited 2025 Feb 3.

[42] World Health Organization. Interim recommendations for use of the ChAdOx1-S [recombinant] vaccine against COVID-19 (AstraZeneca COVID-19 vaccine AZD1222 Vaxzevria™, SII COVISHIELD™). Geneva, Switzerland; 2021. Available from: https://www.who.int/publications/i/item/WHO-2019-nCoV-vaccines-SAGE_recommendation-AZD1222-2021.1. Cited 2025 Feb 3.

[43] World Health Organization. Interim recommendations for the use of the Janssen Ad26.COV2.S (COVID-19) vaccine. Geneva, Switzerland; 2021. Available from: https://www.who.int/publications/i/item/WHO-2019-nCoV-vaccines-SAGE-recommendation-Ad26.COV2.S-2021.1. Cited 2025 Feb 3.

[44] World Health Organization. WHO SAGE roadmap for prioritizing uses of COVID-19 vaccines in the context of limited supply. 2021. Available from: https://www.who.int/publications-detail-redirect/whosage-roadmap-for-prioritizing-uses-of-covid-19-vaccines-in-thecontext-of-limited-supply

[45] Ariana News. Vaccine campaign now open for everyone over 18. ATN News. 2021. Available from: https://www.ariananews.af/vaccine-campaign-now-open-for-everyone-over-18/. Cited 2024 Nov 25.

[46] China Bureau of Disease Control and Prevention. Technical Guidelines for New Coronavirus Vaccination (First Edition). 2021. Available from: http://www.nhc.gov.cn/jkj/s3582/202103/c2febfd04fc5498f916b1be080905771.shtml. Cited 2024 Nov 25.

[47] Bradpiece S, Paquette D. Ivory Coast is falling behind its vaccination schedule. Health workers fear thousands of shots could expire. Washington Post. 2021. Available from: https://www.washingtonpost.com/world/2021/04/04/ivory-coast-coronavirus-vaccines-covax-astrazeneca/. Cited 2024 Nov 25.

[48] Al-Shaikh A, Muthu N, Aidyralieva C, Profili MC, Bellizzi S. COVID-19 vaccine roll-out in middle-income countries: Lessons learned from the Jordan experience. Vaccine. 2021;39(34):4769–71. 34281741 10.1016/j.vaccine.2021.06.078 PMC8238651

[49] Ministry of Health Jordan. Coronavirus vaccine question and answer. Ministry of Health Jordan. Available from: https://corona.moh.gov.jo/ar/Coronavirus-Vaccine. Cited 2021 May 10.

[50] Republic of Sierra Leone Ministry of Health and Sanitation. Environmental and social management framework for the additional financing covid-19 emergency preparedness and response project under the covid-19 strategic preparedness and response program (SPRP). 2021. Available from: https://mohs.gov.sl/index.php/download/sl_-covax-esmf-sierra-leone-final_june-22-2021-updated-docx/.

[51] Ministry of Health Syria. The Ministry of Health launches the #National_Vaccination_Campaign_against_Coronavirus, starting from tomorrow, Sunday, until September 16th. Facebook. 2021. Available from: https://www.facebook.com/MinistryOfHealthSYR/photos/a.350396745617648/834809067176411/?type=3.

[52] Francis JR, de Araujo RM, da Silva VO, Lobo S, Coelho D, Mathur A, et al. The response to COVID-19 in Timor-Leste: lessons learnt. BMJ Glob Health. 2023Oct 11;8(10): e013573. 37821115 10.1136/bmjgh-2023-013573 PMC10583031

[53] United Arab Emirates Ministry of Health and Prevention. Vaccines against COVID-19 in the UAE. Vaccines against COVID-19 in the UAE - The Official Portal of the UAE Government. 2021. Available from: https://u.ae/en/information-and-services/justice-safety-and-the-law/handling-the-covid-19-outbreak/vaccines-against-covid-19-in-the-uae.

[54] Terry E, Cartledge S, Damery S, Greenfield S. Factors associated with COVID-19 vaccine intentions during the COVID-19 pandemic; a systematic review and meta-analysis of cross-sectional studies. BMC Public Health. 2022;22(1):1667. 36056325 10.1186/s12889-022-14029-4 PMC9437387

[55] Azanaw J, Endalew M, Zenbaba D, Abera E, Chattu VK. COVID-19 vaccine acceptance and associated factors in 13 African countries: A systematic review and meta-analysis. Front Public Health. 2022;10:1001423. 36761336 10.3389/fpubh.2022.1001423 PMC9903367

[56] Alam Z, Mohamed S, Nauman J, Al-Rifai RH, Ahmed LA, Elbarazi I. Hesitancy toward vaccination against COVID-19: A scoping review of prevalence and associated factors in the Arab world. Hum Vaccines Immunother. 2023;19(2):2245720. 10.1080/21645515.2023.2245720 PMC10443971 37594508

[57] Wang Q, Hu S, Du F, Zang S, Xing Y, Qu Z, et al. Mapping global acceptance and uptake of COVID-19 vaccination: A systematic review and meta-analysis. Commun Med. 2022;2(1):1–10. 36101704 10.1038/s43856-022-00177-6 PMC9465145

[58] Urueña A, Machado R, Cunha J, LópezColmano C, Rancaño C, Kfouri R, et al. Opinions, attitudes and factors related to SARS-CoV-2 vaccine uptake in eight South American countries. Vaccines. 2023;11(11):1660. 38005992 10.3390/vaccines11111660 PMC10675814

[59] Crawshaw AF, Farah Y, Deal A, Rustage K, Hayward SE, Carter J, et al. Defining the determinants of vaccine uptake and undervaccination in migrant populations in Europe to improve routine and COVID-19 vaccine uptake: a systematic review. Lancet Infect Dis. 2022;22(9):e254–66. 35429463 10.1016/S1473-3099(22)00066-4 PMC9007555

[60] Sileo KM, Hirani IM, Luttinen RL, Hayward M, Fleming PJ. A scoping review on gender/sex differences in COVID-19 vaccine intentions and uptake in the United States. Am J Health Promot AJHP. 2023;17:8901171231200778. 10.1177/08901171231200778 PMC10802093 37847250

[61] The COVID-19 Sex-Disaggregated Data Tracker | Global Health 50/50. Available from: https://globalhealth5050.org/the-sex-gender-and-covid-19-project/the-data-tracker/. Cited 2023 Dec 22.

[62] Azami M, Nasirkandy MP, Esmaeili Gouvarchin Ghaleh H, Ranjbar R. COVID-19 vaccine acceptance among pregnant women worldwide: A systematic review and meta-analysis. PloS One. 2022;17(9):e0272273. 36170334 10.1371/journal.pone.0272273 PMC9518917

[63] Galanis P, Vraka I, Siskou O, Konstantakopoulou O, Katsiroumpa A, Kaitelidou D. Uptake of COVID-19 vaccines among pregnant women: a systematic review and meta-analysis. Vaccines. 2022;10(5):766. 35632521 10.3390/vaccines10050766 PMC9145279

[64] Limaye RJ, Paul A, Gur-Arie R, Zavala E, Lee C, Fesshaye B, et al. A socio-ecological exploration to identify factors influencing the COVID-19 vaccine decision-making process among pregnant and lactating women: Findings from Kenya. Vaccine. 2022. Available from: https://www.sciencedirect.com/science/article/pii/S0264410X22013317. Cited 2022 Nov 2. 10.1016/j.vaccine.2022.10.068 PMC9618426 36336529

[65] Limaye RJ, Singh P, Paul A, Fesshaye B, Lee C, Zavala E, et al. COVID-19 vaccine decision-making among pregnant and lactating women in Bangladesh. Vaccine. 2023;41(26):3885–90. 37208208 10.1016/j.vaccine.2023.05.024 PMC10183608

[66] Nassiri-Ansari T, Atuhebwe P, Ayisi AS, Goulding S, Johri M, Allotey P, et al. Shifting gender barriers in immunisation in the COVID-19 pandemic response and beyond. Lancet Lond Engl. 2022;400(10345):24. 10.1016/S0140-6736(22)01189-8 PMC9246460 35780789

