# Supplementary material for: Gender equity and COVID-19 vaccine policies for pregnant people: a global analysis
Source: Int J Equity Health. 2025 May 7;24:127. doi: 10.1186/s12939-025-02497-0 (PMC12060400; doi:10.1186/s12939-025-02497-0)
Supplement: Supplementary file 1 — Supplementary Material 1. [file 12939_2025_2497_MOESM1_ESM.pdf]

Supplementary Material: Gender equity and COVID-19 vaccine policies for pregnant people: A global analysis.

Table of Contents

Supplementary Table 1. Distribution of GGGI scores by policy position on the use of COVID-19 vaccines in pregnancy at three timepoints. 2

Supplementary Table 2. Odds of a restrictive policy position by GGGI score at three time points, unadjusted and adjusted, with outlier removed. 3

Supplementary Table 3. Frequency of country income level by policy position on the use of COVID-19 vaccines in pregnancy at three timepoints. 4

Supplementary Table 4. Frequency of mRNA vaccine availability by policy position on the use of COVID-19 vaccines in pregnancy at three timepoints. 5

Supplementary Table 1. Distribution of GGGI scores by policy position on the use of COVID-19 vaccines in pregnancy at three timepoints.

	GGGI Score							
	Permissive				Restrictive			
Timepoint	n	mean	SD	(95% CI)	n	mean	SD	(95% CI)
6/1/2021	66	0.73	0.067	(0.71, 0.74)	49	0.70	0.065	(0.68, 0.71)
10/15/2021	120	0.72	0.061	(0.71, 0.73)	17	0.66	0.071	(0.62, 0.69)
3/1/2022	134	0.72	0.060	(0.71, 0.73)	8	0.63	0.090	(0.57, 0.70)

Supplementary Table 2. Odds of a restrictive policy position by GGGI score at three time points, unadjusted and adjusted, with outlier removed.

	**6/1/2021**				**10/15/2021**				**3/1/2022**			
	n	cOR	95%CI	p-val	n	cOR	95%CI	p-val	n	cOR	95%CI	p-val
GGGI	113	0.93	0.87, 0.99	0.023	136	0.88	0.80, 0.96	0.006	141	0.86	0.75, 0.98	0.021
	n	aOR[1]	95%CI	p-val	n	aOR	95%CI	p-val	n	aOR	95%CI	p-val
GGGI	113	0.97	0.90, 1.04	0.353	136	0.91	0.82, 1.00	0.066	141	0.87	0.76, 0.99	0.047

[1] Multiple logistic regression adjusted for country income level and mRNA vaccine availability.
cOR: crude odds ratio; aOR: adjusted odds ratio; GGGI: Global Gender Gap Index

Supplementary Table 3. Frequency of country income level by policy position on the use of COVID-19 vaccines in pregnancy at three timepoints.

	6/1/2021				10/15/2021				3/1/2022			
	Permissive		Restrictive		Permissive		Restrictive		Permissive		Restrictive	
Income level	n	%	n	%	n	%	n	%	n	%	n	%
Low	2	50.0	2	50.0	6	54.5	5	45.5	13	81.3	3	18.8
Low middle	9	34.6	17	65.4	29	76.3	9	23.7	37	90.2	4	9.8
Upper middle	18	45.0	22	55.0	45	86.5	7	13.5	50	94.3	3	5.7
High	56	73.7	20	26.3	83	95.4	4	4.6	85	96.6	3	3.4

Supplementary Table 4. Frequency of mRNA vaccine availability by policy position on the use of COVID-19 vaccines in pregnancy at three timepoints.

	6/1/2021				10/15/2021				3/1/2022			
	Permissive		Restrictive		Permissive		Restrictive		Permissive		Restrictive	
mRNA vaccine availability	n	%	n	%	n	%	n	%	n	%	n	%
Available	62	68.1	29	31.9	120	91.6	11	8.4	137	93.8	9	6.2
Unavailable	23	41.8	32	58.2	43	75.4	14	24.6	48	92.3	4	7.7
